# Recovery of β-carotene from pumpkin using switchable natural deep eutectic solvents

**DOI:** 10.1016/j.ultsonch.2021.105638

**Published:** 2021-06-21

**Authors:** Alena Stupar, Vanja Šeregelj, Bernardo Dias Ribeiro, Lato Pezo, Aleksandra Cvetanović, Aleksandra Mišan, Isabel Marrucho

**Affiliations:** a University of Novi Sad, Institute of Food Technology, Bulevar cara Lazara 1, 21000 Novi Sad, Serbia; b University of Novi Sad, Institute of Food Technology, Institute of Food Technology, Bulevar cara Lazara, 121000 Novi Sad, Serbia, Republic of Serbia; c Escola de Quimica, Universidade Federal do Rio de Janeiro, Av Horacio Macedo, CT, Bl.E, 101, 21941598 Rio de Janeiro, Brazil; d Centro de Química Estrutural and Departamento de Engenharia Química, Instituto Superior Tecnico, Universidade de Lisboa, Avenida Rovisco Pais, 1049-001 Lisboa, Portugal; e University of Belgrade, Institute of General and Physical Chemistry, Studentski trg 12/V, 11000 Belgrade, Serbia

**Keywords:** Natural deep eutectic solvents, Switchable NADES, UAE optimization, β-Carotene, Carotenoids

## Abstract

The aim of the present research was to develop green and sustainable extraction procedure for β-carotene recovery from pumpkin. A series of hydrophobic natural deep eutectic solvents (NADESs) based on fatty acids were prepared to establish high extraction efficiency of β-carotene and to increase stability of extracted carotenoids from the pumpkin. To intensify extraction process, NADES composed of C8 and C10 fatty acids (3:1) was selected and coupled with ultrasound assisted extraction. Response surface methodology and artificial neural network model (ANN) model was adopted to analyze significance of extraction parameters demonstrating high prediction levels of the β-carotene yield, experimentally confirming the maximum β-carotene content of 151.41 µg/mL at the optimal process condition. Extracted carotenoids in the optimal NADES extract have shown high stability during the storing period of 180 days. A switchable-hydrophilicity eutectic solvent system has been introduced as a successful way to recover extracted carotenoids from the NADES solvent. It was capable of precipitating 90% of carotenoids present in the extract. The proposed procedure is simple, easily scalable and has minimal impact on operators and the environment.

## Highlights

• Hydrophobic deep eutectic solvents showed to be effective for carotenoids recovery.
• Ultrasound assisted extraction has improved recovery of β-carotene.
• At optima conditions UAE extract of high-quality extract was obtained.
• Carotenoids content, β-carotene, in extract was preserved.
• Solvent polarity switch enabled carotenoids separation from the solvent.

## Graphical abstract

**Figure:**
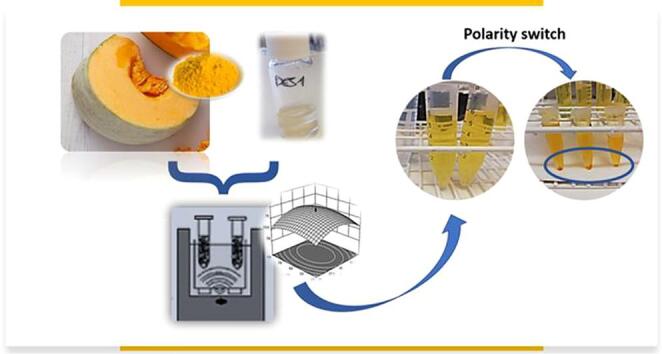

## 1 Introduction

Current trends in food industry direct significant resources to develop new functional food products and modern technologies. By applying functional foods in daily diets, beyond basic nutrition, it contributes to better dietary habit which can manifest positive effects on health by improving overall health conditions and reduce the risk of diseases. The fact that incorporation of highly valuable ingredients in functional foods may increase the content and bioavailability of natural health-promoting compounds and contribute to a better health, resulted in growing consumers interests and demands for functional foods which contains natural bioactive ingredients [1]. Therefore, modern sustainable food processing and production industries face the challenge of not only developing environmentally friendly technologies, to foster the development of new applications in food products, but also to be cost effective, reduce waste, and improve utilization of by-products to recover higher amount of bioactive compounds while maintaining the lowest impact on the environment.

Consumption of carotenoids rich foods, especially β-carotene, has been recognized for its preventive effect on the occurrence and development of many diseases including protection against cancer, cardiovascular diseases and macular degeneration [2], [3]. Additional advantage of β-carotene recovered from natural sources is that it may contribute to additional health improvement due to the presence of supplemental carotenoids simultaneously isolated [4].Therefore, attention is focused on the utilization of underutilized agriculture products rich in carotenoids and food-processing by-products, that could be potentially extracted, purified, and exploited as functional ingredients in value-added products.

Pumpkin (Cucurbita maxima) presents relatively inexpensive and abundant plant material which can be converted into high-value extracts, rich in carotenoids (β-carotene, lutein, zeaxanthin, β-cryptoxanthin), with benefits to both consumers and industry [5]. It is one of the most grown vegetables in agriculture around the globe with 22.9 million tons produced in 2019, growing 2.5% in average each year, in which according to FAOSTAT (2020) China and Russia represent near 50% of world production. However, recovery of pumpkins bioactives falls into one of the main problems of food industry; to find the best way to apply nutrients with limited solubility and bioavailability into food products [6]. The main carotenoid in pumpkin, β-carotene, is characterized by poor solubility in water and low temperature oil, sensitive to light, oxygen, and heat, thus making it difficult to be recovered in high quantities, and incorporated in food products [7]. Selection of the appropriate extraction technology and optimization of the extraction parameters plays a major role in obtaining extracts with the high bioactive content and their further applicability. Presence of diverse sets of carotenoids in matrix with different polarity makes their extraction and separation challenging task [8]. Traditional methods of recovering bioactive molecules include the use of a large amount of plant material, processes that take place at high temperature and/or over a long period of time, use of a large amount of non-selective, toxic and non-environmentally friendly organic solvents. Usual extraction processes for carotenoids recovery involve several organic solvents such as hexane, ethyl acetate, ethanol and their various mixtures, while in case of β-carotene, hexane has been one of the most widely used solvents in the industry, due to its high affinity, however it remains the problem of the elimination of the residual solvent to obtain a safe extract [9]. Therefore, the search for efficient extraction techniques, using natural biocompatible and safe solvents, which simultaneously meet the requirements of sustainable industry is mandatory. Developing a simple, rapid, and efficient extraction methodology for of β-carotene recovery, in particular, and carotenoids, in general, could boost their utilization in functional foods.

Specific attention has been focused on finding innovative extraction techniques which, besides being highly efficient, are sustainable, minimize solvent volume, reduce process time, and are cost effective. In particular, deep eutectic solvents (DES) hold a promising perspective in the alternative solvents scenario, since they can be carefully designed by choosing compounds with appropriate chemical structures, which are translated into important specific properties essential for the extraction of the target compound, while also displaying low environmental impact and toxicity [9]. It is important to emphasize the possibility of adjusting physicochemical properties by simple combination of different salts and hydrogen bond donors, their molar ratio, water content and temperature [10]. Despite the large number of DES that have been recently proposed, researchers are significantly focused on developing new strategies to improve process design and product production [11]. Therefore, the search for compounds from natural sources to prepare DES has revealed an exciting way to overcome the drawback of the common solvents and find the application of natural DES (NADES) in the food industry. Since NADES are task specific solvents, it is possible to tune their properties to attain a high solubilization capacity for specific compounds, such as poorly water-soluble compounds, high extraction and high stabilization ability for some natural products [12]. These characteristics make NADES promising solvents for extraction of β-carotene. In addition, the accessibility of β-carotene is highly dependent on polarity of solvent/carrier, therefore by selecting an appropriate extraction solvent, may results in enhanced biopharmaceutical properties of the extracted β-carotene. NADES as a solvents are often combined with ultrasound assisted extraction (UAE), one of the most efficient, cheapest and simplest existing extraction systems, which could be applied on an industrial level [13], [14]. In this sense combination of green, sustainable, and biodegradable solvents, NADES, with high efficiency extraction technique, UAE, is of a great interest as it can increase penetration of the solvent induced by the ultrasonic cavitation. Combining NADES with UAE, extraction of bioactive compounds is raised to the higher level as it is very effective in decreasing the processing time, reducing the cost of extraction, preventing thermal damage, and enhancing food quality [15], [16].

In order to develop efficient method for carotenoids, precisely β-carotene extraction, this work was focused on the development of an efficient, sustainable solvents (NADES) that could combine high extraction efficiency and increased stabilization of carotenoids. The study presents a new approach based on the potential of NADES based on saturated fatty acids as task specific solvents combined with ultrasound assisted extraction to intensify of the extraction process and increase the recovery of β-carotene from pumpkin. Natural deep eutectic solvents have already found wide application as solvents for recovery of bioactive compounds due to negligible environmental impact and simple and inexpensive preparation, but the greatest challenge remains in recovery of extracted bioactive compounds from the eutectic solvents [10]. Consequently, a novel and elegant approach based on switchable hydrophobic NADES was developed to separate the β-carotene from NADES based pumpkin extract. Additionally, Response Surface Methodology (RSM) and Artificial Neural Network (ANN) were used in the prediction of the β-carotene yield during the extraction, and to characterize the effects of extraction temperature, matrix to solvent ration and ultrasonic power.

## 2 Experimental section

### 2.1 Chemicals

DL-menthol (purity ≥ 95%), octanoic acid (purity ≥ 98%), nonanoic acid (purity ≥ 98%), decanoic acid (purity ≥ 98%), dodecanoic acid (purity ≥ 98%) were purchased from Sigma-Aldrich and used as (purity ≥ 98%) and received in the preparation of hydrophobic deep eutectic solvents. Methanol (99.8%), acetone (99.8%) and hexane (≥99%) of chromatographic grade was obtained from Sigma Aldrich and ultrapure water was obtained from a Milli-Q system (Millipore, Billerica, MA, USA).

### 2.2 Solvent and sample preparation

The pumpkin (*Cucurbita maxima)* was bought on the local market. Chopped pumpkin was freeze-drying using CHRIST Freeze Dryer Alpha 2–4 LD plus lyophilizer (Osterode am Harz, Germany) for 48 h. The lyophilized sample was further pulverized in a Knifetec 1095 Sample Mill (FOSS, Germany). The dominant particle fraction (92%) from 200 to 400 µm with the moisture content of 3.76% was obtained by sieve analysis, using a vibrating shaker sieve set (AS200, Retsch, Germany). Sample was stored in plastic containers until further analysis at −20 °C.

The components for obtaining NADES were mixed in certain ratio (Table 2), and slowly mixed under mild heating at 50 °C. Mixing was performed until complete homogenization and thereafter obtained NADESs were slowly cooled to room temperature.

**Table 1:** Experimental domain with natural and coded values of independent variables used in central composite design.

Independent variable	Factor levels				
	−α	−1	0	1	α
Temperature (°C)	20	30	45	60	70
US Power (%)	30	40	60	80	90
Solvent to solid ratio (g/mL)	7	10	15	20	23

**Table 2:** Apparent solubility and extraction efficiency of β-carotene in the selected NADES measured by spectrophotometric method.

NADES	Composition	β-carotene solubility (µg/mL)	Extraction efficiency of β-carotene from pumpkin (µg/mL)
Caprylic acid: Capric acid	C8:C10 (2:1)	179.44 ± 0.32	93.17 ± 0.92
Caprylic acid: Capric acid	C8:C10 (3:1)	200.77 ± 0.42	96.74 ± 1.03
Caprylic acid: Capric acid	C8:C10 (4:1)	136.61 ± 0.49	77.81 ± 1.05
Caprylic acid: Lauric acid	C8:C12 (3:1)	149.81 ± 0.37	81.52 ± 0.96
Pelargonic acid: Lauric acid	C9:C12 (3:1)	152.75 ± 0.62	81.90 ± 1.55
Capric acid: Lauric acid	C10:C12 (2:1)	148.42 ± 0.56	83.46 ± 1.26
Pelargonic acid: Capric acid: Lauric acid	C9:C10:C12 (3:1:1)	191.66 ± 0.54	83.66 ± 2.09
DL-menthol: Capric acid	M:C10 (2:1)	180.22 ± 0.59	90.33 ± 1.15
DL-menthol: Caprylic acid	M:C8 (1:1)	172.94 ± 0.62	89.49 ± 1.03
DL-menthol: Lauric acid	M:C12 (2:1)	97.18 ± 0.49	81.41 ± 0.98

### 2.3 Screening of hydrophobic NADES for β-carotene extraction

To select a suitable hydrophobic NADES for the extraction of carotenoids from pumpkin, the apparent solubility of β-carotene in different NADES was evaluated. For that purpose, excessive amount of β-carotene was added to the different solvents at room temperature until a saturated solution, solid-liquid equilibria, was achieved. A sample of the saturated solution was centrifuged (Eppendorf minispin, Eppendorf, Germany) for 20 min at room temperature and 5000 rpm. Unsolved β-carotene was removed by filtration (Millex, Germany) and the β-carotene solubility was determined spectrophotometrically [17]. All experiments were replicated three times.

### 2.4 Extraction of carotenoids from pumpkin

To select the best hydrophobic NADES for the extraction of β-carotene from pumpkin, extraction capacities of selected NADES were evaluated by extracting 100 mg of pumpkin sample using different NADES as extraction solvent (1:10). Extractions were performed in three replicates at room temperature, for 24 h and 450 rpm shaking speed on Thermomixer incubator-shaker (Eppendorf, Germany). Extracted carotenoids were expressed as β-carotene (mg/mL).

Conventional solid-liquid extraction was also performed with hexane, until the pumpkin residue became colourless according to the procedure proposed by Šeregelj and co-workers (2017) [18]. This extract was used as a control sample to compare the extraction efficiency of hydrophobic NADES and that of conventional solvents. All experiments were replicated three times.

## 3 Ultrasound assisted extraction (UAE)

To provide intensification of extraction process and recovery of β-carotene from pumpkin, NADES was combined with UAE. To carry out UAE, C8:C10 (3:1) NADES was selected as it showed to have best affinity and solubility of β-carotene. The UAE was conducted in an ultrasound water bath (Elmasonic P30 Elma Hans Schmidbauer GMBH, Germany) with fixed frequency at 37 kHz, previously optimized by Altemimi and co-workers (2015) [16].

### 3.1 Carotenoids analysis

Quantitative analyses of total carotenoids were performed using a spectrophotometric method (UV 1800, Shimadzu, Japan) at 450 nm. The β-carotene concentration of each extract was determined based on a β-carotene standard calibration curve. Calibration curves of β-carotene standards dissolved in suitable solvent were constructed by plotting absorbance vs concentration.

For HPLC analysis of carotenoids, a Shimadzu Prominence chromatography system with SPD-20AV UV–Vis detector (Shimadzu, Japan) was used. Chromatography was performed in solvent gradient by varying the proportion of solvent A (20% (v/v) water and 80% (v/v) methanol) and solvent B (50% (v/v) acetone and 50% (v/v) methanol) at flow rates of 1 mL/min with the following gradient profile: 25% B 0–3 min; 75% B 3–6 min; 90% B 6–10 min; 100% B 10–18 min; 50% B 18–25 min; 25% B 25–32 min, according to the procedure previously described by Šeregelj et al., 2019 [19]. Analyzed samples were diluted in hexane and identified by matching the retention time and spectral characteristics against those of standards.

### 3.2 Stability tests

The effect of storage time, sunlight exposure and temperature on the stability of carotenoids rich extracts in NADES were investigated under the light (25 °C) and under the night (25 and 4 °C). Extract was analysed for the β-carotene concentration at time zero, while aliquots were removed periodically for β-carotene content analysis. For that purpose, total carotenoids content was determined by the previously described spectrophotometric method (2.5).

A first-order kinetics law was hypothesized and calculated to determine the stability of carotenoids at different storage conditions and the kinetic of carotenoids extract degradation was determined by method described by Song et al. (2018) [20].

### 3.3 Carotenoids recovery from NADES extract by switching NADES polarity

The potential of fatty acids to act as switchable solvents was used to isolate β-carotene from NADES based extract. Particularly, ammonium hydroxide (25% NH_4_OH) was used in appropriate molar ratio (4 mol of NH_4_OH:1 mol of C8:C10 (3:1)) to react with fatty acid based NADES. Ammonium hydroxide was added to NADES extract and exhaustively mixed to obtain a single hydrophilic phase through in situ solvent switching. Extract with switched hydrophilic NADES phase was first immersed in an ice-water bath for 2 min and then placed in the dark for 48 h. Afterwards, samples were subjected to the centrifugation (Minispin, Eppendorf, Germany), precipitated carotenoids were separated from liquid phase and analysed spectrophotometrically and by HPLC. Carotenoids that remained in the solvent were also analysed.

### 3.4 Experimental design and statistical analysis

#### 3.4.1 Response surface methodology, RSM

In UAE experimental setup, extraction parameters that influence extraction efficiency of target component as are extraction temperature (20–70 °C), matrix to solvent ratio (7–23 mg/mL) and ultrasonic power (30–90%) were optimized at five levels (Table 1). The parameters and levels were chosen based on preliminary experiments. Extraction time was set to 10 min, previously detected as a time to reach extraction equilibrium. The response variables were fitted to a second-order polynomial model to describe relationship between the responses and the independent variables. Experimental data were analysed using Design Expert v. 12 Trial (State-Ease, USA).

#### 3.4.2 Artificial neural network modelling, ANN

The prediction of β-carotene during the extraction from pumpkin was also investigated, using a feed forward ANN, applying Multilayer Perceptron Model (MLP) which consisted of three layers (input, hidden and output). Before the calculation, input and output data were normalized to improve the behaviour of the ANN [21]. The experimental database for ANN was randomly divided into training, cross-validation and testing data (60%, 20% and 20% of experimental data, respectively). The ANN was trained using the Levenberg–Marquardt algorithm, while the Broyden-Fletcher-Goldfarb-Shanno (BFGS) algorithm was used as an iterative method for solving unconstrained in nonlinear optimization problems. The optimum number of hidden neurons was chosen, and the coefficients associated with the hidden and the output layer were calculated upon minimizing the difference between predicted ANN values and desired outputs, using *R^2^* during testing as a performance indicator [22]. Accuracy of the ANN model was verified by method described by Arsenović, Pezo, Stanković, & Radojević (2015) [23].

## 4 Results and discussion

Extracts represent one of the most interesting forms of recovered bioactive compounds and simplify further application of extracted bioactives in the final product. Wide range of bioactive compounds present in pumpkin make it a good choice for the extract production. Innovative contemporary extractions processes include application of the most modern extraction techniques economically competitive, in accordance with the principles of “green” chemistry [24]. In that sense, ultrasound assisted extraction methods was applied and optimized to enable maximum utilization of bioactive compounds from pumpkin and creation of extracts of high quality.

### 4.1 Selection of NADES

Recently, hydrophobic DES show to be highly effective extractants for the extraction of poorly water soluble compounds [25], [26]. In this work, safe compounds that could be further used as adjuncts in food were chosen to prepare NADES for carotenoids extraction. The sustainable and biocompatible hydrophobic solvents composed of mostly combination of fatty acids or D,L-menthol and fatty acids prepared in this work are shown in Table 2.

The solubility of β-carotene in examined NADES was as follow: C8:C10 (3:1) > C9:C10:C12 (3:1:1) > M:C10 (2:1) ≈ C8:C10 (2:1) > M:C8 (1:1) > C9:C12 (3:1) ≈ C8:C12 (3:1) ≈ C10:C12 (2:1) > C8:C10 (4:1) > M:C12 (2:1). The highest solubility of the target molecule, β-carotene, was observed when the C8:C10 (3:1) NADES was used as extractant, closely followed by C9:C10:C12 (3:1:1), and then by M:C10 (2:1) and C8:C10 (2:1), while others NADES showed notably lower solubility. All the obtained results, except for M:C12 (2:1), clearly show superior solubility of targeted β-carotene when compared to the results obtained by n-hexane (107 µg/mL), the most used solvent for the carotenoids recovery.

Prepared NADES exhibit very good extraction efficiencies of carotenoids recovered from pumpkin, with the dominant β-carotene. The best NADES, reaching extraction efficiencies higher than 90 µg β-carotene /mL, can be rated according to the following order C8:C10 (3:1) > C8:C10 (2:1) > M:C10 (2:1), which agrees with the solubility order. Accordingly, it can be concluded that these three eutectic solvents present the best performance for the extraction of carotenoids from pumpkin. Although DL-menthol based NADES had already been showed to be good solvents for carotenoids extraction [27], in this work better results were achieved using fatty acid based NADES. This could be explained by the polarity of dominant carotenoids in pumpkin (β-carotene-nonpolar, β-cryptoxanthin and lutein even more polar) [28]. Some results showed that xanthophylls as is lutein, having polar ionone rings, have different interaction with the membrane lipids then β-carotene [2]. Therefore, xanthophylls (lutein) present in pumpkin in smaller amounts, could interact with carboxyl groups from fatty acids eutectics, better than menthol-based eutectics. Additionally, solvent physico-chemical characteristics are directly related to the extraction efficiency of the target compounds and could influence the extraction efficiency [29].

Based on all the prominent positive properties of selected NADES, primarily their “green” origin, easy preparation and effective solubility and extraction efficiency of carotenoids, and especially β-carotene, these solvents can be used for extraction of carotenoids from other food and food by-products. Therefore, the most prominent NADES, C8:C10 (3:1), was chosen for the further optimization using CCD with RSM and ANN modelling. Due to the fact that medium-chain fatty acids can also be considered as switchable hydrophobic solvent [30], this solvent can be further switched between hydrophobic and hydrophilic forms by the adjustment of pH of the solution, providing a good recovery method for hydrophobic solutes, such as β-carotene.

### 4.2 Optimization of DES and extraction condition

#### 4.2.1 RSM and ANN model fitting

The choice of extraction technique and its parameters have a crucial role in the successful recovery of bioactive compounds as the optimization of the experimental parameters reflects on the possible implementation at industrial level and influences the costs and efficiency of process. RSM and ANN are the most frequently used approaches for extraction process optimization. The experimental data used for modelling and optimization included three independent variables (solvent to solid ratio, temperature, and ultrasound power), to reach the highest β-carotene content in pumpkin extract.

**Fig. 1:**
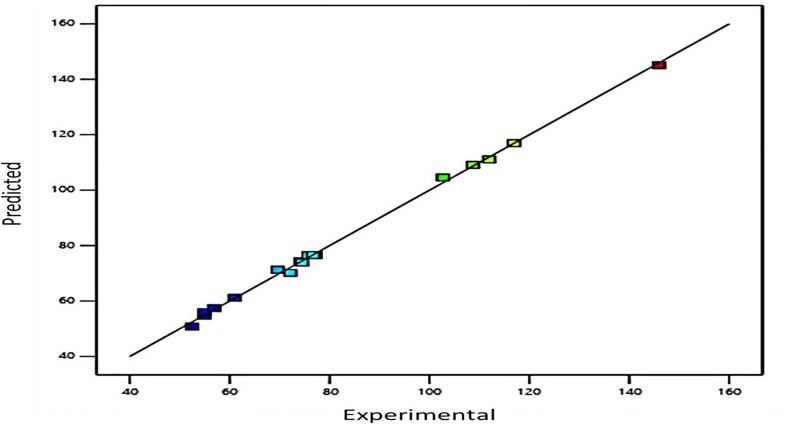
Experimental measured and RSM predicted values of β-carotene content during the extraction.

The regression coefficients, the model for investigated response, the correspondent β-carotene content, and the results of the ANOVA are presented in Table 3. Statistical analysis showed that the proposed model is adequate in accordance with the Tukey's HSD (*p* ≤ 0.05) and that it has no significant lack of fit (*p* > 0.05). Descriptive statistics parameters such as *R^2^* and adjusted *R^2^* and *CV* provided additional information about model fitness. The tested responses can be explained by the proposed regression equation with the obtained value *R^2^* of 0.99. Additionally, the *R^2^* and adjusted *R^2^* of the developed model implicated great conformity between the experimental and predicted values. Reproducibility of the models was good, with *CV* of 2.32%. Determination of regression coefficients using MLS can provide model equations for UAE of carotenoids extraction from pumpkin, which is able to predict response values within investigated experimental domain (Table 3). The predicted experimental variable (β-carotene content) match well according to low dispersion of points presented in Fig. 1.

**Table 3:** Analysis of variance (ANOVA) for second-order polynomial model for investigated β-carotene content.

Source	Degrees of freedom	Sum of Squares	F-value	p-value	Coefficient estimation
**Model**	9	111.84	505.3	< 0.0001	
Intercept					76.7
A-Temperature (°C)	1	0.016	0.649	0.447	0.34
B-US Power (%)	1	0.0034	0.1364	0.7228	0.1461
C-Solvent to solid ratio (mg/mL)	1	98.28	3996.09	< 0.0001	−27.39
AB	1	0.0089	0.3631	0.5658	0.2953
AC	1	0.8461	34.4	0.0006	−3.22
BC	1	0.4726	19.22	0.0032	−2.17
A^2^	1	0.5142	20.91	0.0026	−2.07
B^2^	1	0.0998	4.06	0.0838	−0.8643
C^2^	1	9.38	381.35	< 0.0001	9.62
**Residual**	7	0.1721			
Lack of Fit	5	0.1631	7.17	0.1268	
Pure Error	2	0.0091			
**Cor.Total**	16	112.01

The ANN procedure of StatSoft Statistica (Statistica, 2010) was used to model the ANN, and the number of hidden neurons was 9, there were 3 inputs and 1 output (network MLP 3–9-1), when obtaining high values of R^2^ (0 0.984; 0 0.999 and 1 0.000 for training, testing and validation performances, respectively) and low values of SOS (Table 4). A multi-layer perceptron model consisting of three layers (input, hidden and output) was used for model establishment. This model has been proven as quite capable of approximating nonlinear functions [22].

**Table 4:** The “goodness of fit” tests for the developed ANN model.

Network name	Performance			Error			Train. algorith.	Error func.	Hidden active.	Output active.
	Train.	Test.	Valid.	Train.	Test.	Valid.				
MLP 3-9-1	0.984	0.999	1.000	13.516	3.815	9.818	BFGS 12	SOS	Tanh	Tanh

*Performance terms represent the coefficients of determination, while error terms indicate a lack of data for the ANN model.

**Fig. 2:**
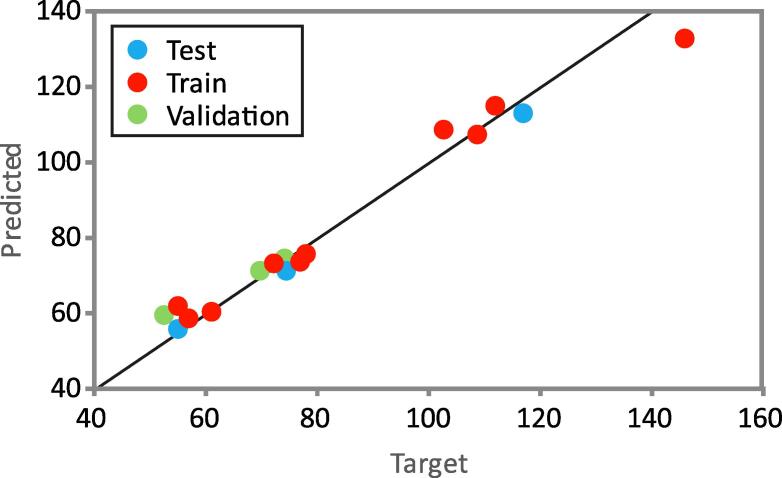
Experimental measured and ANN model predicted values of β-carotene content during the extraction.

The ANN model predicted the experimental variable (β-carotene content) match reasonably well according to relatively low dispersion of points from the diagonal line for a wide range of the process variables, as seen in Fig. 2, where the experimentally measured and ANN model predicted values of β-carotene content were presented.

The quality of the model fit was tested, and the residual analysis of the developed model was presented in Table 4. The ANN model had an insignificant lack of fit tests, which means the model satisfactorily predicted carotenoids extraction. A high *R^2^* is indicative that the variation was accounted for and that the data fitted the proposed model satisfactorily [31].

The “goodness of fit” tests for the developed ANN model can be evaluated through the values of *R*^2^, *χ*^2^, *RMSE* and *MPE*, 0.973 23.696, 4.723 and 4.489, respectively, showing that the developed ANN model was adequate for experimental results prediction. The mean of residuals for ANN model for the yield of β-carotene content prediction was −0.099, the standard deviations was 4.867, while the skewness and the kurtosis were 0.792 and 1.991, respectively. These results demonstrated a good approximation to a normal distribution around zero with a probability of 95% (2 × SD), which means a good generalization ability of ANN model for the range of observed experimental values [32].

#### 4.2.2 Influence of investigated extraction parameters

According to the previously presented results, the C8:C10 (3:1) NADES showed the best performance in carotenoids extraction, therefore it was selected for experimental design. The efficiency of extraction is strongly dependent on several experimental extraction parameters so temperature, US power and solvent to solid ratio were investigated. A total of 17 experimental points were investigated and listed in Table 5. Recovered carotenoids content from pumpkin may vary depending upon cultivars and variety, but it can also change in wide range depending on the applied extraction technique and the experimental conditions, which was also noticed in this research, since the β-carotene yield greatly changed, from 52.5 up to 145.94 (µg/mL), depending on the different experimental extraction conditions.

**Table 5:** Five-level, three-variable experimental design applied for UAE and experimentally observed values of investigated response in terms of carotenoids content expressed as b-carotene.

Run	Temperature (°C)	US power (%)	Solvent to solid ratio (mL/g)	Concentration of β- carotene (µg/mL)
1	20.0	60.0	15	72.14
2	30.0	80.0	20	54.95
3	30.0	40.0	10	102.71
4	30.0	40.0	20	61.01
5	30.0	80.0	10	108.70
6	45.0	60.0	15	76.70
7	45.0	90.0	15	74.15
8	45.0	60.0	15	77.14
9	45.0	30.0	15	74.47
10	45.0	60.0	23	56.89
11	45.0	60.0	7	145.94
12	45.0	60.0	15	75.82
13	60.0	40.0	10	111.93
14	60.0	40.0	20	54.90
15	60.0	80.0	20	52.50
16	60.0	80.0	10	116.94
17	70.0	60.0	15	69.59

Influence of investigated UAE conditions towards extraction of β-carotene was expressed according to p-values (significant if p ≤ 0.05) for the regression coefficients in the second-order polynomial model presented in Table 3. According to data from Table 3 and Table 5, the most significant effect was expressed by linear and quadratic terms of solvent to solid ratio, and by the interaction of this parameter with temperature and US power. One of the most important parameters to be optimized in an extraction process in order to efficient use of energy and production of minimal amount of waste is matrix to solvent ratio, as it influences the energy consumption and affect production of waste amount. Contemporary trends in green extractions strive to the minimization of solvent use, which goes along with our results as the target volumes of NADES did not increase efficiencies because of the dilution effect.

**Fig. 3:**
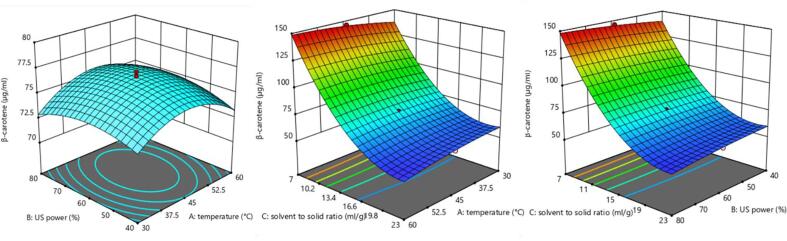
Response surface plots showing the combined effects of the extraction parameters on β-carotene extraction.

The quadratic term of temperature and interaction of temperature and solvent to solid ratio show to be significant (p ≤ 0.05), while the linear term of temperature was not significant. According to Fig. 3, despite the β-carotene thermal instability, increase in temperature did not affect extraction nor degradation of β-carotene, probably due to short extraction time. This suggests temperature range is in the correlation with the research of Sun and co-workers (2011) when extraction of β-carotene increased significantly with increasing of temperature from 25 to 45 °C and the research Purohit and co-workers (2015) who have selected temperature of 50 °C as optimum for UAE extraction for β-carotene from carrot waste [33], [34]. Obtained results indicate that mild conditions can be used to gain similar yield but saving the time and energy.

The UAE regarded as “green” extraction technique has been already used for the extraction of carotenoids from different sources showing the high extraction efficiency, but as the effect of ultrasound cavitation may accelerate or trigger unwanted chemical reactions in the extraction medium, the ultrasonic power must be investigated as one of the most important parameters in UAE. Interaction of temperature with mild heating showed to have a significant impact on β-carotene extraction as the ultrasonication combined with mild heating results in a disruption of cell walls due to the acoustic cavitation phenomenon causing the release of cellular contents and breaking intermolecular interactions, speeding up extraction process [35].

#### 4.2.3 Optimization of the β-carotene yield

The optimal parameters for β-carotene maximization were extraction temperature of 50 °C, ultrasonic power of 60% (52.5 W/cm^3^) and solvent to solid ration of 7 mL/g during the 10 min for carotenoids extraction from pumpkin reaching β-carotene content of 151.41 ug/mL. By choosing the low temperature (50 °C) and short extraction time it is avoided high oxidative degradation and isomerization of carotenoids and the generation of free radicals which can be triggered by high temperatures and cavitation [27]. Selected condition should be easily applicable with the low-cost demands also on the others food sources or food by-products rich in carotenoids, precisely of β-carotene.

**Fig. 4:**
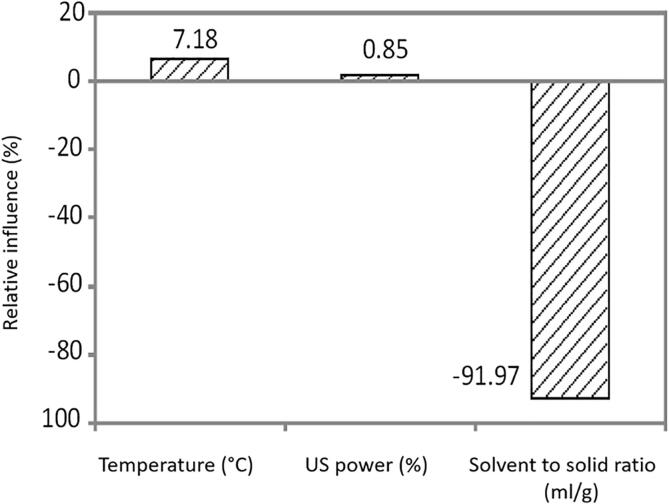
The relative importance of the parameters on β-carotene yield, determined using Yoon interpretation method.

Optimization of the extraction process of β-carotene from pumpkin was also performed using the developed ANN model, by maximizing its output, to ensure the highest yield and the optimal processing conditions, with the high product quality and a high throughput capacity. The optimum process conditions for β-carotene extraction were as follows: temperature was equal to 60 °C, US power was 60% (52.5 W/cm^3^), and solid to solvent ratio was 7 mL/g. According to the ANN results, the maximum β-carotene content was 135.39 µg/mL. The adequacy of the developed ANN model was determined by performing two independent experiments at the gained process conditions to obtain the minimum and maximum β-carotene content [36]. According to the Fig. 4, the influence of V was the most significant parameter with approximately relative importance of −91.97%%, while the influence of temperature and US power was + 7.18% and + 0.85%, respectively.

### 4.3 Stability of obtained extract

Given the susceptibility of carotenoids to degradation, development of delivery systems with the potential to decrease carotenoid degradation is of high relevance. Several reports indicate that NADES enhances stability of bioactive compounds, where stability of bioactive compounds is related to the hydrogen bonding interactions between solutes and solvent molecules, but also media pH [17], [37]. The stability and bioactivity of NADES based extracts are important factors influencing the suitability of NADES as delivery media. Accordingly, the stability of carotenoids during storage was monitored at different conditions, to test the potential of NADES to serve as a vehicle for carotenoids.

Carotenoids content decreased more rapidly at room temperature when extract was exposed to light. During the first month of the storage, degradation of 21% occurred for the extract exposed to the light at room temperature, while for the extract kept the at the same temperature in the dark, this reduction was about 15%. The carotenoids degradation followed the first-order kinetic with a good data fit, and the corresponding *R^2^* values (0.96 and 0.83) for the samples stored at room temperature. Rate constants for light and dark storage conditions were 0.0086 and 0.0034 day^−1^, while half-life values were 80.58 and 203.82 days, respectively. The sample exposed to the light, degraded faster than in the dark, probably due to isomerization and oxidation processes. The extract kept in dark, in the refrigerator, was minimally degraded (2.2%) during the first month of examination, while total reduction of 7.3% occurred during the period of 6 months of storage. Referring to the obtained results, pumpkin carotenoids are stable in NADES as a solvent if kept in dark, at 4 °C can be preserved for at least 6 months.

There are several reports indicating that NADES enhances stability of bioactive compounds [17], [37], [38], which was also confirmed in this research. According to literature, stability of bioactive compounds can be related to the hydrogen bonding interactions between solutes and solvent molecules, but also pH may affect the stability of bioactive molecules as are carotenoids [37], [38].

### 4.4 Recovery of compounds from NADES extracts by switching NADES polarity

An interesting approach has been proposed by Jessop and co-workers (2010) through the use of a switchable-hydrophilicity solvent, where solvent removal from a hydrophobic product could be achieved be reversibly switching solvent polarity, from hydrophobic to hydrophilic [39], while Sed and co-workers (2018) described, for the first time, switchable NADES-based solvents, where the switching serves the purpose to separate the compound of interest or fraction it from the solvent [40]. Based on switchable solvent with the reversible transformation between hydrophilicity and hydrophobicity we propose the sustainable separation of carotenoids from hydrophobic NADES extract based on possibility of switchable potential of fatty acids based NADES to precipitate carotenoids from the carotenoids rich extract.

**Fig. 5:**
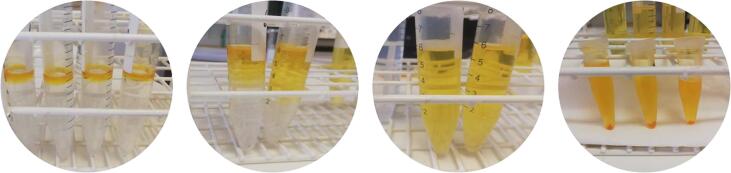
Extract polarity switching from hydrophobic to hydrophilic and carotenoids precipitation as results of polarity switch: a) water addition to the hydrophobic NADES extract causing the formation of two phases; b) change in the polarity of NADES extract; c) single phase formation after brief vortex stirring; d) precipitation of carotenoids in a polar phase.

By adding the water, to the NADES extract (Fig. 5, a) caused the formation of two phases because of different polarities of mixed media, however adding weak base as is NH_4_OH to the extract (Fig. 5, b), the pH of the extract was changed, which caused the change in the polarity of NADES extract, causing two initial phases start mixing. After the addition of sufficient amount of NH_4_OH and brief vortex stirring, only one phase was observed which was stable over the time (Fig. 5, c). By obtaining a single, hydrophilic phase it was demonstrated that the fatty acid based NADES can be switched in situ. Further steps of the experiment present extract after the polarity switching and 48 h, when precipitation of the carotenoids occurred on the bottom of the vial (Fig. 5, d) as a result of their low solubility in the changed polarity media. The precipitated carotenoids can be separated by simple decantation or additional filtration. This switching procedure is fast and requires a minimum intervention of external apparatus or complex lab equipment and can be applied for carotenoid recovery in the case the oily, hydrophobic extract rich in carotenoids is not suitable for a targeted product or pure carotenoids are preferred. After the separation of remaining solvent from precipitated carotenoids, remaining solvent could be further switched back to hydrophobic by pumping CO_2_ into the remaining solvent-extract [41]. Another possibility is to use it in a hydrophilic form, obtained after switching, for the extraction of hydrophilic molecules from the pumpkin. In this way both polar and non-polar compounds could be successively extracted and separated.

### 4.5 HPLC analysis of carotenoids in optimal UAE extract, supernatant and precipitate carotenoids after extract polarity switching

**Fig. 6:**
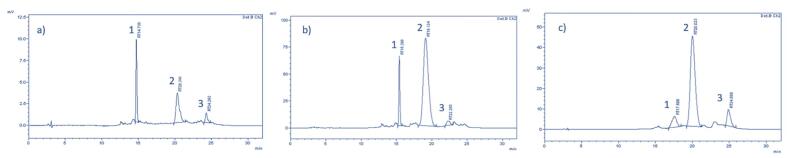
HPLC analysis of a) extract, b) supernatant and c) crystals with peaks of lutein (1), β-carotene (2) and β-cryptoxantin (3).

In analysed samples, extract, supernatant after polarity switching, and precipitate, three most dominant carotenoids, lutein, β-carotene and β-cryptoxanthin were detected. β-carotene was the most dominant carotenoid in all fractions, followed by β-cryptoxanthin in extract and precipitate, while supernatant was slightly more abundant in lutein then β-cryptoxanthin. Referring to HPLC analysis, three dominant carotenoids were detected in the extract, lutein (1.88 ug/mL), β-carotene (70.22 ug/mL) and β-cryptoxanthin (69.77 ug/mL) (Fig. 6, a). After the extract polarity switching, significant reduction (of 90%) of carotenoids was obtained in supernatant. Dominant carotenoid in supernatant was β-carotene, its significant reduction of 86% was obtained comparing to the extract. Significant reduction of 97% was also observed in β-cryptoxanthin content in supernatant. However, reduction of lutein in supernatant was negligible. Accordingly, precipitated fraction was dominant in β-carotene (52.25%), followed by β-cryptoxanthin (38.04%) while the least abundant was lutein. The obtained results are consistent with the polarities of molecules and solvents. After the switch from the hydrophobic media to hydrophilic, β-carotene and β-cryptoxanthin, which are nonpolar molecules, precipitated, while lutein, more polar molecule then β-carotene and β-cryptoxanthin, remained in soluble form.

## 5 Conclusion

Obtained results provide valuable information for carotenoids recovery from the pumpkin and possible utilization of carotenoids rich NADES extract in functional food products. Fatty acids based NADES, C8:C10 (3:1), showed the highest solubility of the target molecule, β-carotene, and good stability of recovered compound in extract over the time. Combining the UAE and fatty based NADES, extraction process was intensified and resulted in extract rich in β-carotene content. Applied extraction method showed to be a promising environmentally friendly extraction method from the point of view of green chemistry, using effective solvent in lower amount and parallel use of low energy inputs. As NADES are non-toxic and biodegradable do not affect the quality or safety of the extract, the purification step could be eliminated, and carotenoids rich extract could be directly applied in food or products which make it competitive and feasible for industrial application. However, when pure bioactive compounds are emphasized, a switchable, hydrophilic solvent-based method which was demonstrated in this research to be used for separation of carotenoids from NADES based extract. This study provided a practical example demonstrating the efficiency of hydrophobic NADESs as designer solvent to extract carotenoids from pumpkin and by switching the polarity, carotenoids were separated from the solvent in an elegant way with minimal impact on operators and the environment which represents an innovative step forward in relation to existing technologies.

## CRediT authorship contribution statement

**Alena Stupar:** Conceptualization, Investigation, Writing - original draft, Writing - review & editing. **Vanja Šeregelj:** Investigation. **Bernardo Dias Ribeiro:** Methodology. **Lato Pezo:** Formal analysis. **Aleksandra Cvetanović:** Validation, Visualization. **Aleksandra Mišan:** Supervision, Resources, Funding acquisition. **Isabel Marrucho:** Conceptualization, Supervision, Resources.

## Declaration of Competing Interest

The authors declare that they have no known competing financial interests or personal relationships that could have appeared to influence the work reported in this paper.

## References

[1] McClements D.J., Zou L., Zhang R., Salvia-Trujillo L., Kumosani T., Xiao H. (2015). Enhancing Nutraceutical performance using excipient foods: designing food structures and compositions to increase bioavailability. Compr. Rev. Food Sci. Food Saf..

[2] Augustynska D., Jemioła-Rzemińska M., Burda K., Strzałka K. (2015). Influence of polar and nonpolar carotenoids on structural and adhesive properties of model membranes. Chem. Biol. Interact..

[3] Arvayo-Enríquez H., Mondaca-Fernández I., Gortárez-Moroyoqui P., López-Cervantes J., Rodríguez-Ramírez R. (2013). Carotenoids extraction and quantification: a review. Anal. Methods.

[4] Ribeiro B.D., Barreto D.W., Coelho M.A.Z. (2011). Technological aspects of β-carotene production. Food Bioprocess. Technol..

[5] Shi X., Wu H., Shi J., Xue S.J., Wang D., Wang W., Cheng A., Gong Z., Chen X., Wang C. (2013). Effect of modifier on the composition and antioxidant activity of carotenoid extracts from pumpkin (Cucurbita maxima) by supercritical CO2. LWT – Food Sci. Technol..

[6] Vranješ M., Tot A., Zhang S. (2019). New liquid components. Encycl. Ion. Liq..

[7] Silva H.D., Cerqueira M.A., Souza B.W.S., Ribeiro C., Avides M.C., Quintas M.A.C., Coimbra J.S.R., Carneiro-da-Cunha M.G., Vicente A.A. (2011). Nanoemulsions of β-carotene using a high-energy emulsification–evaporation technique. J. Food Eng..

[8] Gu Z., Deming C., Yongbin H., Zhigang C., Feirong G.u. (2008). Optimization of carotenoids extraction from Rhodobacter sphaeroides. LWT - Food Sci. Technol..

[9] Ribeiro B.D., Florindo C., Iff L.C., Coelho M.A.Z., Marrucho I.M. (2015). Menthol-based eutectic mixtures: Hydrophobic low viscosity solvents. ACS Sustain. Chem. Eng..

[10] Mišan A., Nađpal J., Stupar A., Pojić M., Mandić A., Verpoorte R., Choi Y.H. (2020). The perspectives of natural deep eutectic solvents in agri-food sector. Crit. Rev. Food Sci. Nutr..

[11] Florindo C., Branco L.C., Marrucho I.M. (2019). Quest for green-solvent design: from hydrophilic to hydrophobic (deep) eutectic solvents. ChemSusChem.

[12] Dai Y., Witkamp G.-J., Verpoorte R., Choi Y.H. (2015). Tailoring properties of natural deep eutectic solvents with water to facilitate their applications. Food Chem..

[13] Radošević K., Bubalo M.C., Srček V.G., Grgas D., Dragičević T.L., Redovniković I.R. (2015). Evaluation of toxicity and biodegradability of choline chloride based deep eutectic solvents. Ecotoxicol. Environ. Saf..

[14] Duan L.i., Dou L.-L., Guo L., Li P., Liu E.-H. (2016). Comprehensive evaluation of deep eutectic solvents in extraction of bioactive natural products. Sustain. Chem. Eng..

[15] Chemat F., Rombaut N., Sicaire A.-G., Meullemiestre A., Fabiano-Tixier A.-S., Abert-Vian M. (2017). Ultrasound assisted extraction of food and natural products. Mechanisms, techniques, combinations, protocols and applications. A review. Ultrason. Sonochem..

[16] Altemimi A., Lightfoot D., Kinsel M., Watson D. (2015). Employing response surface methodology for the optimization of ultrasound assisted extraction of lutein and β-carotene from spinach. Molecules.

[17] Jeliński T., Przybyłek M., Cysewski P. (2019). Natural deep eutectic solvents as agents for improving solubility, stability and delivery of curcumin. Pharm. Res..

[18] Seregelj V., Cetkovic G., Canadanovic-Brunet J., Tumbas-Saponjac V., Vulic J., Stajcic S. (2017). Extraction and encapuslation of bioactive compounds from carrots. Acta Period. Technol..

[19] Šeregelj V., Tumbas Šaponjac V., Lević S., Kalušević A., Ćetković G., Čanadanović-Brunet J., Nedović V., Stajčić S., Vulić J., Vidaković A. (2019). Application of encapsulated natural bioactive compounds from red pepper waste in yogurt. J. Microencapsul..

[20] Song H.Y., Moon T.W., Choi S.J. (2019). Impact of antioxidant on the stability of β-carotene in model beverage emulsions: role of emulsion interfacial membrane. Food Chem..

[21] Grieu S., Faugeroux O., Traoré A., Claudet B., Bodnar J.-L. (2011). Artificial intelligence tools and inverse methods for estimating the thermal diffusivity of building materials. Energy Build..

[22] Chattopadhyay P.B., Rangarajan R. (2014). Application of ANN in sketching spatial nonlinearity of unconfined aquifer in agricultural basin. Agric. Water Manag..

[23] Arsenović M., Pezo L., Stanković S., Radojević Z. (2015). Factor space differentiation of brick clays according to mineral content: prediction of final brick product quality. Appl. Clay Sci..

[24] Zengin G., Cvetanović A., Gašić U., Stupar A., Bulut G., Şenkardes I., Dogan A., Ibrahime Sinan K., Uysal S., Aumeeruddy-Elalfi Z., Aktumsek A., Fawzi Mahomoodally M. (2020). Modern and traditional extraction techniques affect chemical composition and bioactivity of Tanacetum parthenium (L.) Sch. Bip. Ind. Crops Prod..

[25] D.J.G.P. van Osch, Design and applications of hydrophobic deep eutectic solvents, 2018. https://pure.tue.nl/ws/portalfiles/portal/103474989/20180918_Osch.pdf.

[26] Martins Mónia.A.R., Crespo E.A., Pontes P.V.A., Silva L.P., Bülow M., Maximo G.J., Batista E.A.C., Held C., Pinho Simão.P., Coutinho João.A.P. (2018). Tunable hydrophobic eutectic solvents based on terpenes and monocarboxylic acids. ACS Sustain. Chem. Eng..

[27] Ricarte G.N., Coelho M.A.Z., Marrucho I.M., Ribeiro B.D. (2020). Enzyme-assisted extraction of carotenoids and phenolic compounds from sunflower wastes using green solvents. 3 Biotech..

[28] Kreck M., Kürbel P., Ludwig M., Paschold P.J., Dietrich H. (2006). Identification and quantification of carotenoids in pumpkin cultivars (Cucurbita maxima L.) and their juices by liquid chromatography with ultraviolet-diode array detection. J. Appl. Bot. Food Qual..

[29] Zannou O., Koca I. (2020). Optimization and stabilization of the antioxidant properties from Alkanet (Alkanna tinctoria) with natural deep eutectic solvents. Arab. J. Chem..

[30] Vakh C., Pochivalov A., Andruch V., Moskvin L., Bulatov A. (2016). A fully automated effervescence-assisted switchable solvent-based liquid phase microextraction procedure: Liquid chromatographic determination of ofloxacin in human urine samples. Anal. Chim. Acta.

[31] Turányi T., Tomlin A.S. (2014). Analysis of Kinetic Reaction Mechanisms, Springer. Berlin Heidelberg.

[32] Taylor B.J. (2006). Methods and procedures for the verification and validation of artificial neural networks, Springer. US.

[33] Purohit A.J., Gogate P.R. (2015). Ultrasound-assisted extraction of β-carotene from waste carrot residue: effect of operating parameters and type of ultrasonic irradiation. Sep. Sci. Technol..

[34] Mukherjee P.K., Maity N., Nema N.K., Sarkar B.K. (2011). Bioactive compounds from natural resources against skin aging. Phytomedicine.

[35] Tomšik A., Pavlić B., Vladić J., Ramić M., Brindza J., Vidović S. (2016). Optimization of ultrasound-assisted extraction of bioactive compounds from wild garlic (Allium ursinum L.). Ultrason. Sonochem..

[36] Madamba P.S. (2002). The response surface methodology: an application to optimize dehydration operations of selected agricultural crops. LWT - Food Sci. Technol..

[37] Dai Y., Rozema E., Verpoorte R., Choi Y.H. (2016). Application of natural deep eutectic solvents to the extraction of anthocyanins from Catharanthus roseus with high extractability and stability replacing conventional organic solvents. J. Chromatogr. A.

[38] Dai Y., Verpoorte R., Choi Y.H. (2014). Natural deep eutectic solvents providing enhanced stability of natural colorants from safflower (Carthamus tinctorius). Food Chem..

[39] Jessop P.G., Phan L., Carrier A., Robinson S., Dürr C.J., Harjani J.R. (2010). A solvent having switchable hydrophilicity. Green Chem..

[40] Sed G., Cicci A., Jessop P.G., Bravi M. (2018). A novel switchable-hydrophilicity, natural deep eutectic solvent (NaDES)-based system for bio-safe biorefinery. RSC Adv..

[41] Cicci A., Sed G., Jessop P.G., Bravi M. (2018). Circular extraction: an innovative use of switchable solvents for the biomass biorefinery. Green Chem..

